# Oral immunization with a probiotic cholera vaccine induces broad protective immunity against *Vibrio cholerae* colonization and disease in mice

**DOI:** 10.1371/journal.pntd.0007417

**Published:** 2019-05-31

**Authors:** Brandon Sit, Ting Zhang, Bolutife Fakoya, Aklima Akter, Rajib Biswas, Edward T. Ryan, Matthew K. Waldor

**Affiliations:** 1 Division of Infectious Diseases, Brigham & Women’s Hospital, Boston, Massachusetts, United States of America; 2 Department of Microbiology, Harvard Medical School, Boston, Massachusetts, United States of America; 3 Division of Infectious Diseases, Massachusetts General Hospital, Boston, Massachusetts, United States of America; 4 Department of Medicine, Harvard Medical School, Boston, Massachusetts, United States of America; 5 Department of Immunology and Infectious Diseases, Harvard T.H. Chan School of Public Health, Boston, Massachusetts, United States of America; 6 Howard Hughes Medical Institute, Bethesda, Maryland, United States of America; Johns Hopkins University Bloomberg School of Public Health, UNITED STATES

## Abstract

Oral cholera vaccines (OCVs) are being increasingly employed, but current killed formulations generally require multiple doses and lack efficacy in young children. We recently developed a new live-attenuated OCV candidate (HaitiV) derived from a *Vibrio cholerae* strain isolated during the 2010 Haiti cholera epidemic. HaitiV exhibited an unexpected probiotic-like activity in infant rabbits, preventing intestinal colonization and disease by wild-type *V*. *cholerae* before the onset of adaptive immunity. However, it remained unknown whether HaitiV would behave similarly to other OCVs to stimulate adaptive immunity against *V*. *cholerae*. Here, we orally immunized adult germ-free female mice to test HaitiV’s immunogenicity. HaitiV safely and stably colonized vaccinated mice and induced known adaptive immune correlates of cholera protection within 14 days of administration. Pups born to immunized mice were protected against lethal challenges of both homologous and heterologous *V*. *cholerae* strains. Cross-fostering experiments revealed that protection was not dependent on vaccine colonization in or transmission to the pups. These findings demonstrate the protective immunogenicity of HaitiV and support its development as a new tool for limiting cholera.

## Author summary

Oral cholera vaccines are increasingly used as public health tools for prevention of cholera and curtailing the spread of outbreaks. However, current killed vaccines provide minimal protection in young children, who are especially susceptible to this diarrheal disease, and require ~7–14 days between vaccination and development of protective immunity. We recently created HaitiV, a live-attenuated oral cholera vaccine candidate derived from a clinical isolate from the Haiti cholera outbreak. Unexpectedly, HaitiV protected against cholera-like illness in infant rabbits within 24 hours of administration, before the onset of adaptive immunity. However, HaitiV’s capacity to stimulate adaptive immune responses against the cholera pathogen were not investigated. Here, we report that HaitiV induces immunological correlates of protection against cholera in adult germ-free mice and leads to protection against disease in their offspring. Protection against disease was transferable through the milk of the immunized mice and was not due to transmission or colonization of HaitiV in this model. Coupling the immunogenicity data presented here with our previous observation that HaitiV can protect from cholera prior to the induction of adaptive immunity, we propose that HaitiV may provide both rapid-onset short-term protection from disease while eliciting stable and long-lasting immunity against cholera.

## Introduction

The bacterial pathogen *Vibrio cholerae* causes the severe human diarrheal disease cholera, a potentially fatal illness characterized by rapid-onset of fluid loss and dehydration. Recent estimates place the global burden of cholera at ~3 million cases per year, and over 1.3 billion people are at risk of this disease [1]. *V*. *cholerae* proliferates in the small intestine and produces cholera toxin (CT), which leads to water and electrolyte secretion into the intestinal lumen [2].

The O1 serogroup of *V*. *cholerae* causes virtually all epidemic cholera. This serogroup includes two serotypes, Inaba and Ogawa, whose LPS structures differ by a single methyl group on the terminal O-antigen sugar [3]. Serologic and epidemiologic studies have established the existence of extensive serotype cross-reactivity and -protectivity, although immunogenicity and protection is highest to the homologous serotype [4–7]. Toxigenic O1 strains are divided into two major biotypes, classical and El Tor, but the former has not been isolated in over a decade and is thought to be extinct [8]. Ongoing evolution of El Tor *V*. *cholerae* has given rise to variant El Tor strains, which are distinguishable from earlier strains by a variety of features, including the expression of non-canonical *ctxB* alleles that may impact disease severity in afflicted patients [4,9,10]. These contemporary strains, such as the *ctxB7-*expressing *V*. *cholerae* strain responsible for the 2010 Haitian cholera epidemic, are thought to be the globally dominant cause of cholera [10–12]. Currently, serogroup O139 isolates only cause sporadic disease [13]. Notably, antibodies (or immune responses) targeting the O1 O-antigen do not protect against O139 challenge and vice versa [14–16].

Oral cholera vaccines (OCVs) have recently become widely accepted as a tool for cholera control [17]. Vaccines are a potent method to combat cholera due to their ability to both directly and indirectly reduce disease and transmission [18]. Killed multivalent whole-cell OCVs, such as Shanchol, have shown promise both to prevent disease in endemic regions and as reactive agents to limit cholera during epidemics [19]. However, killed OCVs tend to be less effective at eliciting protective immunity in young children (<5 years old), who are most susceptible to cholera [20,21]. Additionally, these vaccines typically require two doses over the span of several weeks, although recent studies suggest that a single dose may still lead to moderate protection [20,22,23].

There is no live-attenuated OCV licensed for use in cholera-endemic regions. The only clinically available live-attenuated OCV is Vaxchora (CVD103-HgR), which is derived from a classical O1 Inaba *V*. *cholerae* strain and was approved by the US FDA in 2017 for use in travelers [24]. In contrast to killed OCVs, live vaccines, such as CVD103-HgR and the El Tor-derived vaccine Peru-15, elicit more potent immune responses in young children [25,26], potentially because they more closely mimic natural infection than killed OCVs. In particular, live vaccines can produce antigens in vivo that are not expressed in the in vitro growth conditions used to prepare killed vaccines; furthermore, the inactivation processes used to formulate killed vaccines can destroy antigenic epitopes [27].

In addition to the requirement for multiple doses of some OCVs for optimal protection, all current live and killed OCVs are thought to be accompanied by a post-vaccination lag in protection during induction of anti-*V*. *cholerae* adaptive immunity. The shortest reported time to protective efficacy is 8 days post-vaccination, a delay that could hamper reactive vaccination campaigns designed to limit the spread of cholera outbreaks [28]. We recently created HaitiV, a new live-attenuated OCV candidate derived from a variant El Tor O1 Ogawa *V*. *cholerae* clinical isolate from the 2010 Haiti cholera outbreak. HaitiV harbors many genetic alterations that render it avirulent and resistant to reversion while preserving its robust capacity for colonization of the small intestine [29]. In an infant rabbit model of cholera [30], intestinal colonization with HaitiV conferred protection against lethal wild-type (WT) *V*. *cholerae* challenge within 24 hours of vaccination, a timescale inconsistent with the development of adaptive immunity and suggestive of a “probiotic”-like mechanism of protection. Here, using a mouse model of *V*. *cholerae* intestinal colonization, we show that oral administration of HaitiV to female mice elicits serum vibriocidal antibodies and protects their pups from lethal challenge with virulent *V*. *cholerae*. Thus, HaitiV has the potential to provide rapid probiotic-like protection as well as to elicit long-lasting immune protection from cholera.

## Methods

### Bacterial strains and growth conditions

All bacteria were grown in Luria-Bertani (LB) broth supplemented with the relevant chemicals at the following concentrations: streptomycin (Sm, 200μg/mL), kanamycin (Km, 200μg/mL), carbenicillin (Cb, 50μg/mL), sulfamethoxazole/trimethoprim (SXT, 80 and 16μg/mL) and 5-bromo-4-chloro-3-indolyl-β-d-galactopyranoside (X-gal, 60μg/mL). For growth on plates, LB + 1.5% agar was used. All *V*. *cholerae* strains in this study, except for PIC158 and PIC018, were spontaneous Sm^R^ derivatives of the wild-type. Bacteria were stored as -80°C stocks in LB with 35% glycerol. S1 Table lists the strains used in this study.

### Construction of Δ*ctxAB* Haiti *V*. *cholerae*

The CT deletion strain in the H1 *V*. *cholerae* background (HaitiWT) was generated by allelic exchange as previously described, with an additional selection step to enhance the efficiency of obtaining a stable single crossover strain [29]. Briefly, HaitiWT was conjugated with SM10λpir *E*. *coli* bearing the suicide plasmid pCVD442-ctxAB-KmR, containing *sacB* as well as a kanamycin resistance cassette from pKD4 sandwiched by homology arms targeting the *ctxAB* operon (locus tags N900_RS07040 –N900_RS07045). Single crossovers were selected on LB+Sm/Cb/Km agar plates. To select for a double crossover, verified single crossovers were grown in LB + Cb/Km for 4 hours at 37°C and then passaged in LB+10% sucrose overnight at room temperature. Sucrose-resistant (*sacB*-negative), Km^R^ and Cb^S^ colonies were then conjugated with SM10λpir *E*. *coli* bearing pCVD442-ctxAB (no Km^R^ cassette) and clean Km^S^ double crossovers generated via an identical protocol. The Δ*ctxAB* deletion was verified by colony PCR with internal and flanking primers.

### Oral immunization regimen

4-week old germ-free (GF) female C57BL/6 (Massachusetts Host-Microbiome Center) or Swiss-Webster (Taconic Farms) mice were housed in a BL-2 facility for the duration of the experiment. On Day 0, 2, 4, 6, 14, 28, 42 and 56, mice were anesthetized with isoflurane and orally gavaged with 10^9^ CFU of an overnight culture of either HaitiV or CVD103-HgR in 100μL 2.5% Na_2_CO_3_. Mice were weighed at every immunization and once every 4–5 days between boosts. At each weighing, fresh fecal pellets were plated on LB + Sm to enumerate shed bacteria. At Day 7, 14, 28 and 42 post first immunization, blood samples were obtained from each mouse by tail vein incision. A Day 1 blood sample was collected from the Swiss-Webster cohort and the single-dose C57BL/6 cohort. Blood was clotted at room temperature for 1 hour, centrifuged at 20000 x g for 5 minutes and the supernatant (serum) stored at -20°C for analysis.

### Quantification of vibriocidal antibody responses

Vibriocidal antibody quantification was performed by complement-mediated cell lysis using PIC018 (Inaba) or PIC158 (Ogawa) *V*. *cholerae* as the target strain as previously described [31]. Seroconversion was defined as ≥4x increase in titer over the baseline measurement. The characterized mouse monoclonal antibody 432A.1G8.G1.H12 targeting *V*. *cholerae* O1 OSP was used as a positive control for the vibriocidal assay. Titers are reported as the dilution of serum causing a 50% reduction in target optical density compared to no serum control wells.

### Quantification of antigen-specific immune responses

Anti-cholera toxin B subunit (CtxB) and anti-O-specific polysaccharide (OSP) responses were measured by previously described isotype-specific ELISAs [32,33]. Briefly, 96-well plates (Nunc) were coated with 1 μg/mL solution of bovine GM1 monosialoganglioside (Sigma) in 50mM carbonate buffer overnight. Next, 1μg/mL CtxB in 0.1% BSA/PBS purified from the classical Inaba strain 569B (List Biological Laboratories) was layered onto the GM1-coated wells. Wells were blocked with a 1% BSA/PBS mixture after which 1:50 dilutions of the mouse serum samples were loaded into each well. Goat anti-mouse IgA, IgG or IgM secondaries conjugated to HRP (Southern Biotechnology) were then added at a concentration of 1μg/mL in 0.1% BSA/0.05% Tween/PBS and incubated for 90 minutes. Detection was performed by adding an ABTS/H_2_O_2_ mixture to the wells and taking an absorbance measurement at 405nm with a Vmax microplate kinetic reader (Molecular Devices Corp., Sunnyvale, CA). Plates were read for 5 min at 30 s intervals, and the maximum slope for an optical density change of 0.2 U was reported as millioptical density units per minute (mOD/min). Results were normalized using pooled control serum from mice previously immunized against *V*. *cholerae* and reported as ELISA Units as previously described [32]. Anti-OSP responses were measured and reported similarly to anti-CtxB responses, only instead of CtxB, purified OSP:BSA from either PIC018 or PIC158 (1 μg/mL) was used to coat plates as previously described [34]. Additionally, OSP ELISAs were carried out with 1:25 dilutions of the serum samples.

### Infant mouse challenge assay

The infant mouse survival challenge was adapted from previous reports to optimize the dosage for HaitiWT and to include more frequent monitoring intervals [31,32]. Pregnant dams were singly housed at E18-19 for delivery. At P3 (third day of life), pups were orally inoculated with 50μL LB containing 10^7^ CFU of a directly diluted 30°C overnight culture of *V*. *cholerae* and returned to their dam. Infected pups were monitored every 4–6 hours for onset of diarrhea and reduced body temperature. Once symptoms appeared, monitoring was increased to every 30 minutes until moribundity was reached, at which point pups were removed from the nest and euthanized by isoflurane inhalation followed by decapitation for dissection and CFU plating of the small intestine on LB + Sm/X-gal. Pups that were alive at 48 hpi were deemed protected from the challenge. Cross-fostering was performed by transferring up to half of a litter between dams on the first day of life (P1). Fostering was maintained for at least 48 hours before infection to fully replace the milk from the original dam. We excluded rejected pups from analyses due to our inability to attribute mortality to infection alone.

### Statistical analysis

Statistical analyses were performed with Prism 8 (Graphpad). To analyze whether immune responses were significantly changed over time, a one-way ANOVA was performed. Due to missing values from paired measurements as a result of insufficient serum sample volumes, antibody titers were analyzed with a mixed-effects model one-way ANOVA using the earliest sample (Day 1 or Day 7) as the control and post hoc tests performed with a Dunnett’s multiple comparison test. Survival curves were analyzed with the log-rank (Mantel-Cox) test and CFU burdens were compared with the Mann Whitney U test. A p-value <0.05 was considered statistically significant.

### Animal use statement

This study was performed in accordance with the NIH Guide for Use and Care of Laboratory animals and was approved by the Brigham and Women’s Hospital IACUC (Protocol #2016N000416). Infant (P14 or younger) mice were euthanized by isoflurane inhalation followed by decapitation. When required, adult mice were euthanized by isoflurane inhalation followed by cervical dislocation.

## Results

### Experimental design and vaccine colonization

While the infant rabbit model enables investigation of the progression of a *V*. *cholerae*-induced diarrheal disease that closely mimics human cholera [30], it is not appropriate to study vaccine immunogenicity because newborn animals lack a fully developed immune system. Instead, we used adult GF mice to study HaitiV immunogenicity. In contrast to normal adult mice, which are resistant to *V*. *cholerae* intestinal colonization, oral inoculation of GF mice with *V*. *cholerae* results in stable intestinal colonization without adverse effects [35–37]. In the GF model, serum markers of immunity, such as vibriocidal titers, can be measured, but challenge studies are not possible due to the persistent colonization of the vaccine strain and the resistance of adult mice to diarrheal disease. Here, we further developed a variation of the GF model [38]. Besides measuring serum markers in the orally vaccinated adult mice, neonatal pups (which are sensitive to *V*. *cholerae* induced diarrheal disease) born to these mice were subjected to challenge studies to evaluate vaccine protective efficacy.

**Fig 1:**
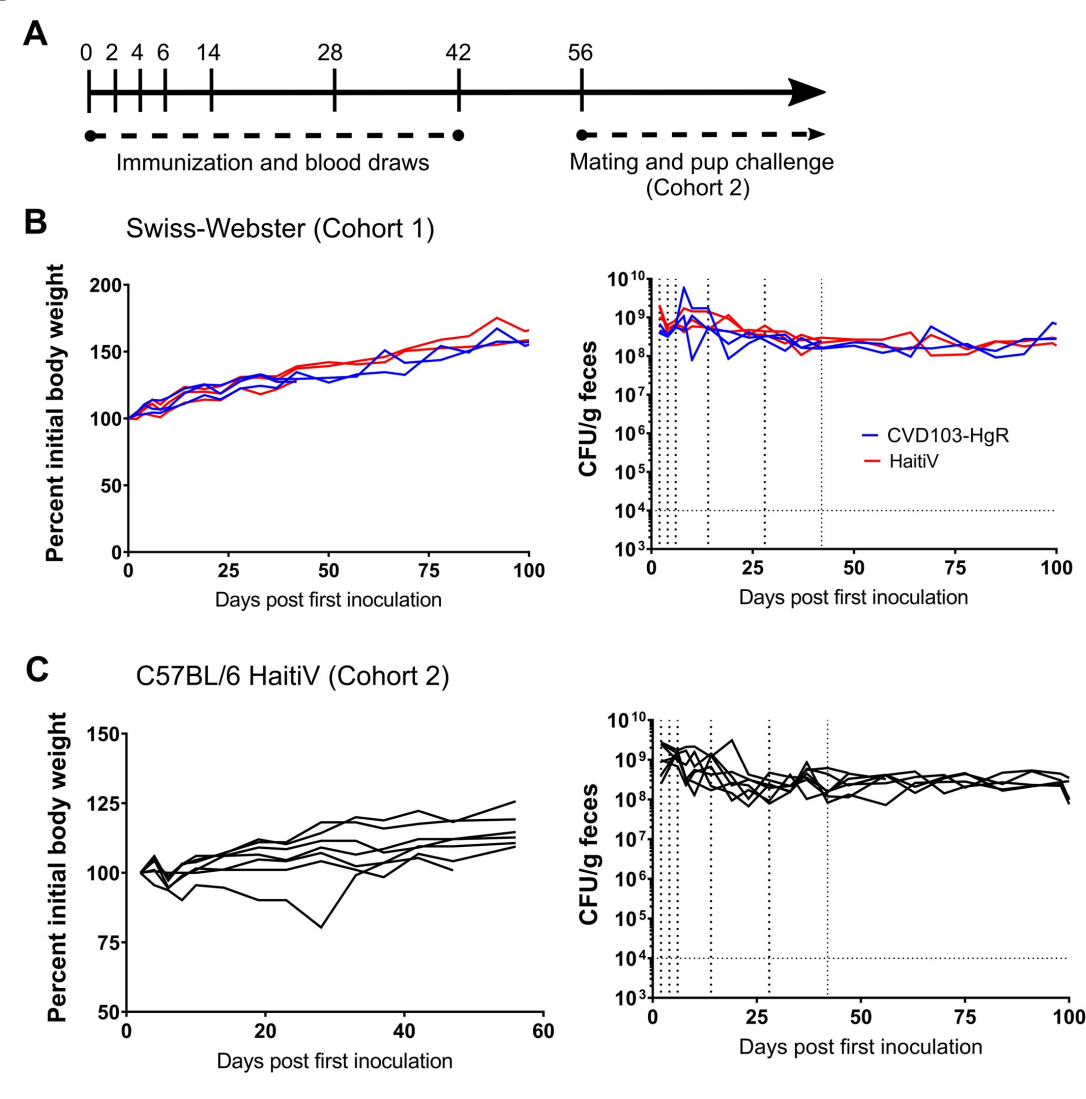
Fecal vaccine shedding and weight of adult GF mice after oral administration of live attenuated cholera vaccines. (A): Schematic of vaccination regime–note that only cohort 2 was used for protection studies. (B and C): Bodyweight (left) and fecal CFU shedding of HaitiV (right) for cohort 1 and 2 respectively. Curves that stop before day 100 represent mice that were lost during the study due to injury resulting from oral gavage or bleeding.

We established two cohorts of orally immunized adult female GF mice. In the first cohort, a small pilot study was set up to compare the immunogenicity of HaitiV and a streptomycin-resistant derivative of CVD-103HgR. This cohort consisted of 4-week-old Swiss-Webster GF mice that were immunized with either vaccine strain (n = 3 per group). Cohort 2 consisted of a set of 4-week-old C57BL/6 mice that were all immunized with HaitiV (n = 7). We generally followed the multi-dose oral immunization scheme previously used in this model, which included eight doses of 1x10^9^ CFU vaccine over eight weeks [36,37]. After this vaccination regimen, the mice in cohort 2 were mated and vaccine-induced protective immunity was assessed in the progeny (Fig 1A).

Based on fecal CFU, all animals in both cohorts were stably colonized with high levels of either vaccine strain (Fig 1B). No adverse effects of long-term colonization with HaitiV or CVD103-HgR were noted, and all mice gained weight over the course of the study (Fig 1B). Fecal shedding and presumably intestinal colonization of HaitiV in cohort 2 was eliminated after these dams were used to cross-foster pups born to specified-pathogen free (SPF) control mice (described below), suggesting that a normal microbiota can outcompete HaitiV.

### HaitiV immunization elicits robust serum antibodies targeting *V*. *cholerae*

**Fig 2:**
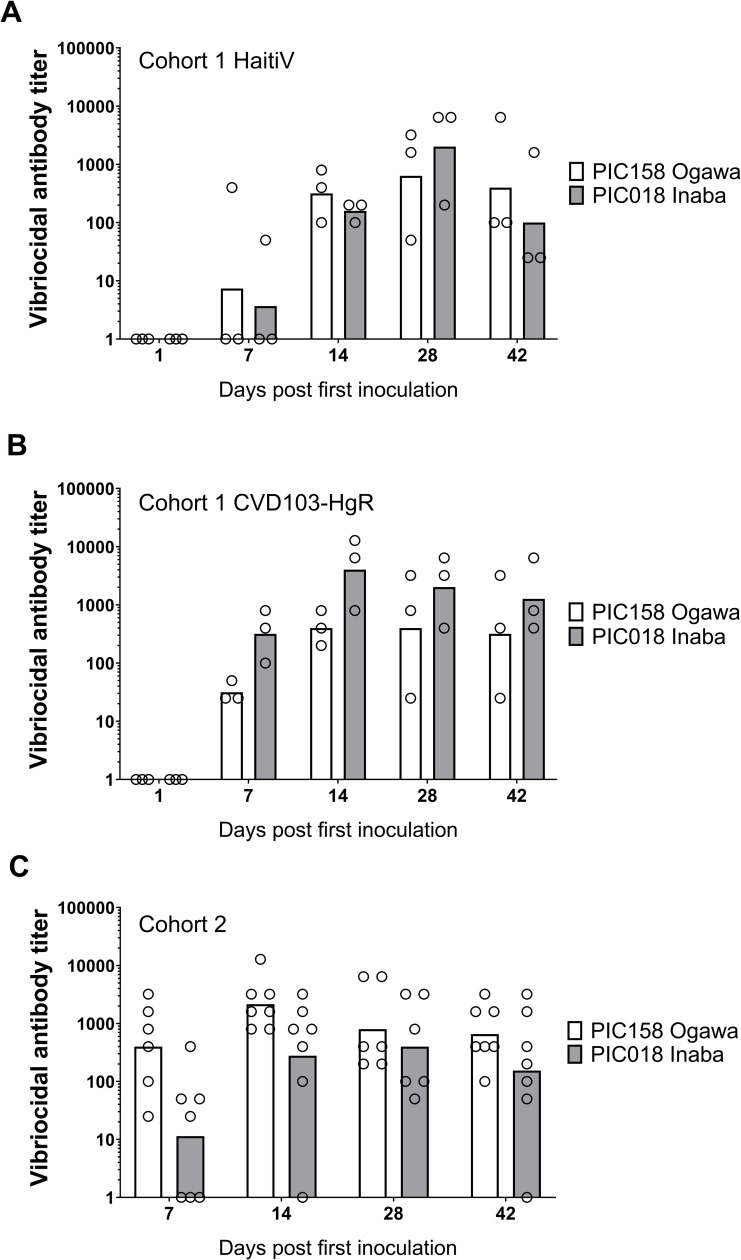
Vibriocidal titers in immunized mice. Vibriocidal titers measured in cohort 1 mice immunized with (A): HaitiV or (B): CVD103-HgR, and in (C): cohort 2 mice immunized with HaitiV are shown. Each circle corresponds to the lowest dilution at which vibriocidal activity was observed and the height of the bars represent geometric mean titers in each group. Ogawa-specific responses, measured using strain PIC158, are shown in white and Inaba-specific responses, measured using strain PIC018, are shown in gray. Serum samples with undetectable vibriocidal activity were assigned a titer of 1.

Serum samples from the immunized mice were used to quantify antibodies targeting several *V*. *cholerae* factors thought to play roles in protection from cholera. One of these metrics, the vibriocidal antibody titer, is a validated correlate of protection in vaccinated humans [39–42]. In cohort 1, all mice immunized with HaitiV or CVD-103HgR seroconverted within 2 weeks and developed vibriocidal titers consistent with those reported in human studies for live OCVs (Fig 2) [41,43]. Furthermore, HaitiV and CVD-103HgR elicited comparable vibriocidal titers.

**Fig 3:**
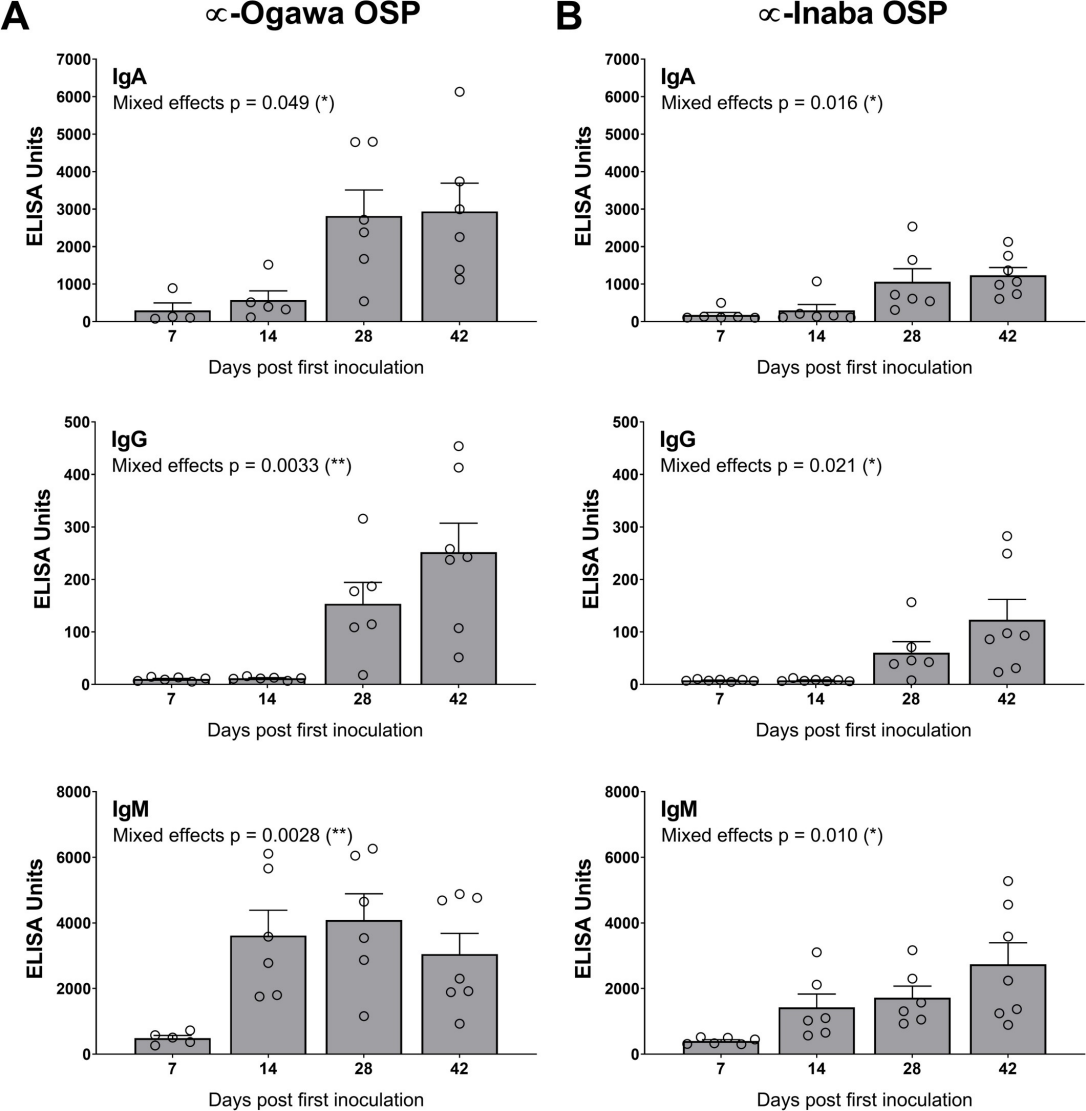
Ogawa- and Inaba-specific anti-OSP titers in C57BL/6 mice immunized with HaitiV. (A) Anti-Ogawa OSP titers with specific isotype data for IgA (top), IgG (middle) and IgM (bottom). (B) Anti-Ogawa OSP titers with specific isotype data for IgA (top), IgG (middle) and IgM (bottom). The mean ± SEM is indicated. Mixed-effect p-values were determined by one-way ANOVA analysis. Multiple comparison p-values for this data are reported in S1 Table. Although there were 7 mice per group, some samples were of insufficient volume for analysis.

**Fig 4:**
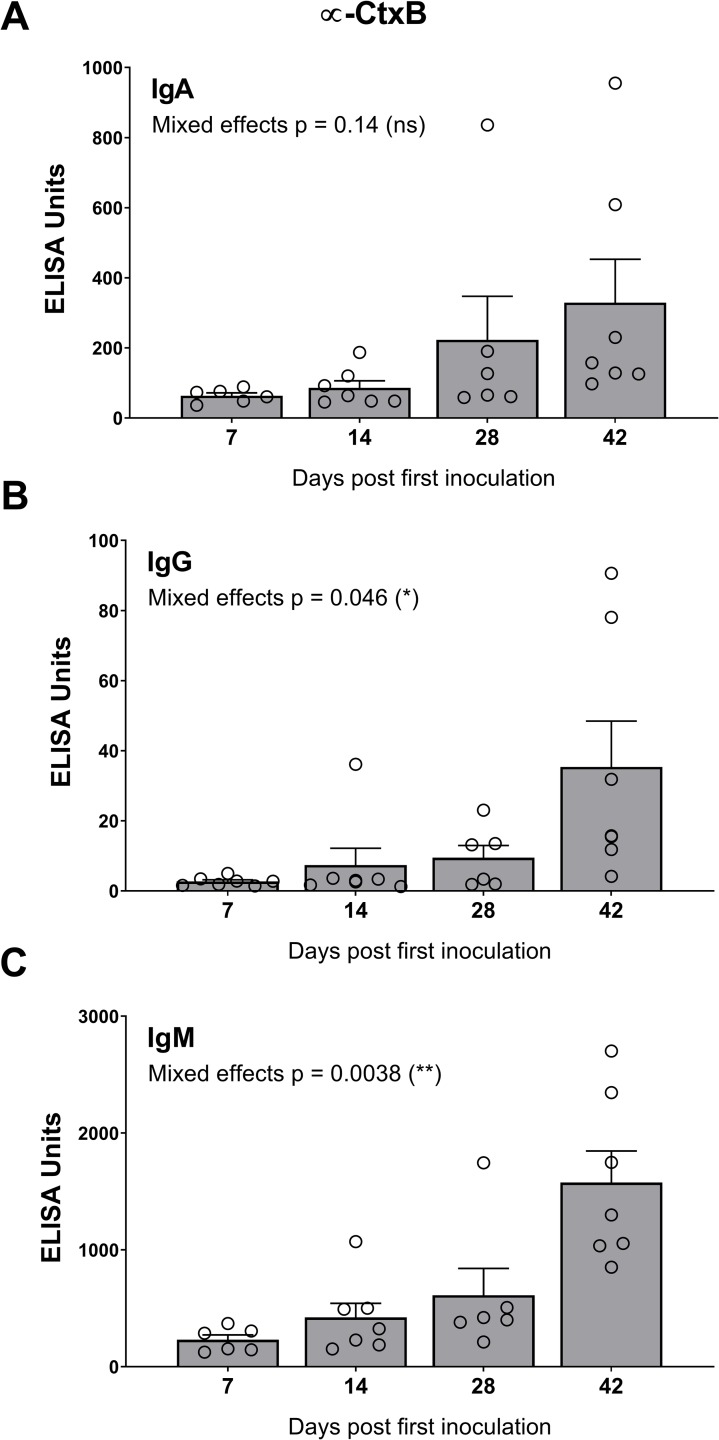
Isotype specific anti-CtxB titers in C57BL/6 mice immunized with HaitiV. Anti-CtxB titers with specific isotype analysis for IgA (A), IgG (B) and IgM (C) were performed for each serum sample. The mean ± SEM is indicated. Mixed-effect p-values were determined by one-way ANOVA analysis. Multiple comparison p-values for this data are reported in S1 Table. Although there were 7 mice per group, some samples were of insufficient volume for analysis.

In cohort 2, HaitiV immunization of C57BL/6 mice also induced high vibriocidal titers to Ogawa and Inaba target strains (Fig 2C). Isotype-specific levels of antibodies targeting Ogawa and Inaba OSP, and CtxB were also measured since they also likely contribute to immunity to cholera [39]. Although we did not measure Day 1 titers in cohort 2, measurements from naïve GF C57BL/6 mice and baseline measurements from cohort 1, and Day 1 of HaitiV-inoculated C57BL/6 mice in a later cohort (S2 Fig) showed undetectable levels of vibriocidal antibodies (Figs 2A and S2). The cohort 2 mice developed strong anti-Ogawa and anti-Inaba OSP responses (Fig 3, S2 Table). The anti-Ogawa OSP titers were generally higher than those targeting Inaba OSP, likely reflecting the fact that HaitiV is an Ogawa strain. All mice in cohort 2 also developed high levels of anti-CtxB IgA, IgG and IgM antibodies (Fig 4, S1 Table). The 100% seroconversion rate and general increase over time of all three humoral immune responses measured (vibriocidal, anti-CtxB and anti-OSP antibodies) reveals that orally delivered HaitiV can elicit *V*. *cholerae*-specific immune responses.

### Pups born to HaitiV immunized dams are protected from lethal *V*. *cholerae* challenge

**Fig 5:**
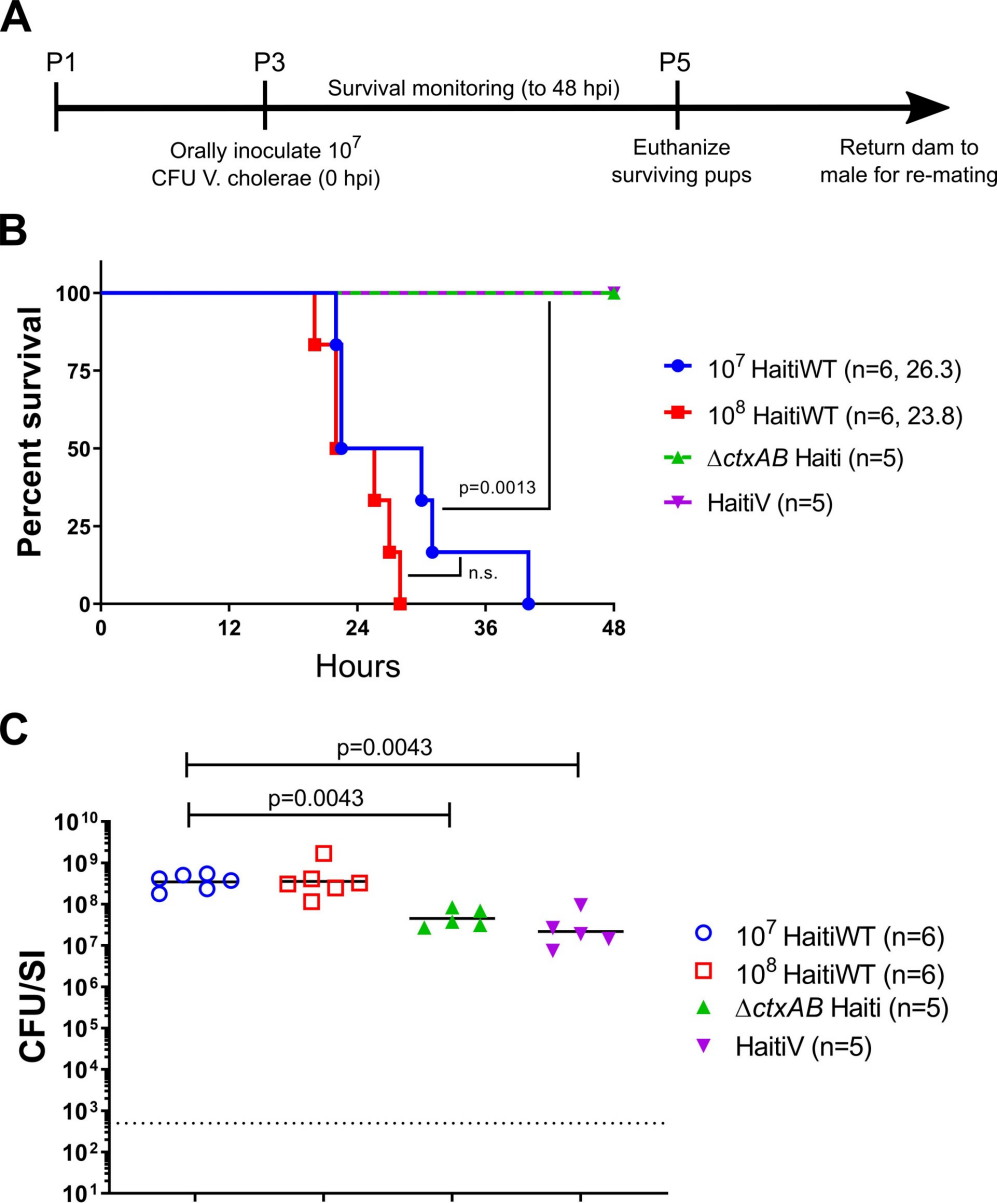
Survival of P3 C57BL/6 pups challenged with Haiti *V*. *cholerae*. (A): Timeline of oral challenge assays. (B) Survival curves for 48 hours post inoculation with the indicated strains; the number of pups challenged and the median survival time in hours is shown. There were no deaths in the Δ*ctxAB* (green) or HaitiV (purple) groups. P-values were determined by the Mantel-Cox method. (C) Small intestinal *V*. *cholerae* burden at the time of death (open shapes) or at 48 hours (filled triangles). Burden is plotted as colony-forming units per small intestine (CFU/SI). The dotted line marks the limit of detection (500 CFU/SI). P-values were determined by the Mann-Whitney U test.

To assess the protective efficacy of HaitiV in this model, we challenged the neonatal progeny of HaitiV-immunized or control dams with lethal doses of different wild type *V*. *cholerae* strains. This assay has been used to study passive immunity elicited by cholera vaccines, but has not been characterized in vaccinated GF mice [31,32,44]. Initially, we optimized this assay with litters from SPF C57BL/6 control mice. Three or four-day old pups were inoculated with 10^7^ or 10^8^ CFU of HaitiWT, the virulent strain from which HaitiV was derived, and returned to their dams for monitoring (Fig 5A). Infected pups from both groups rapidly developed signs of dehydrating diarrheal disease, including accumulation of nest material on their anogenital regions, lethargy, skin tenting and hypothermia. All infected pups died by 48 hours post inoculation (hpi), with a median time to moribundity of ~23–26 hpi (Fig 5B). At the time of death, all pups were heavily colonized, with >10^7^ CFU/small intestine (Fig 5C), and had swollen ceca, another hallmark of productive cholera infection in mammalian models [30,45]. Since there were no significant differences in survival or bacterial loads in mice challenged with either 10^7^ or 10^8^ CFU, the smaller dose was used in subsequent experiments (Fig 5B). Diarrhea and death in this model were entirely dependent on CT; infant mice inoculated with 10^7^ CFU HaitiWT Δ*ctxAB* or HaitiV were completely healthy at 48 hpi, despite sustained intestinal colonization (Fig 5C).

**Fig 6:**
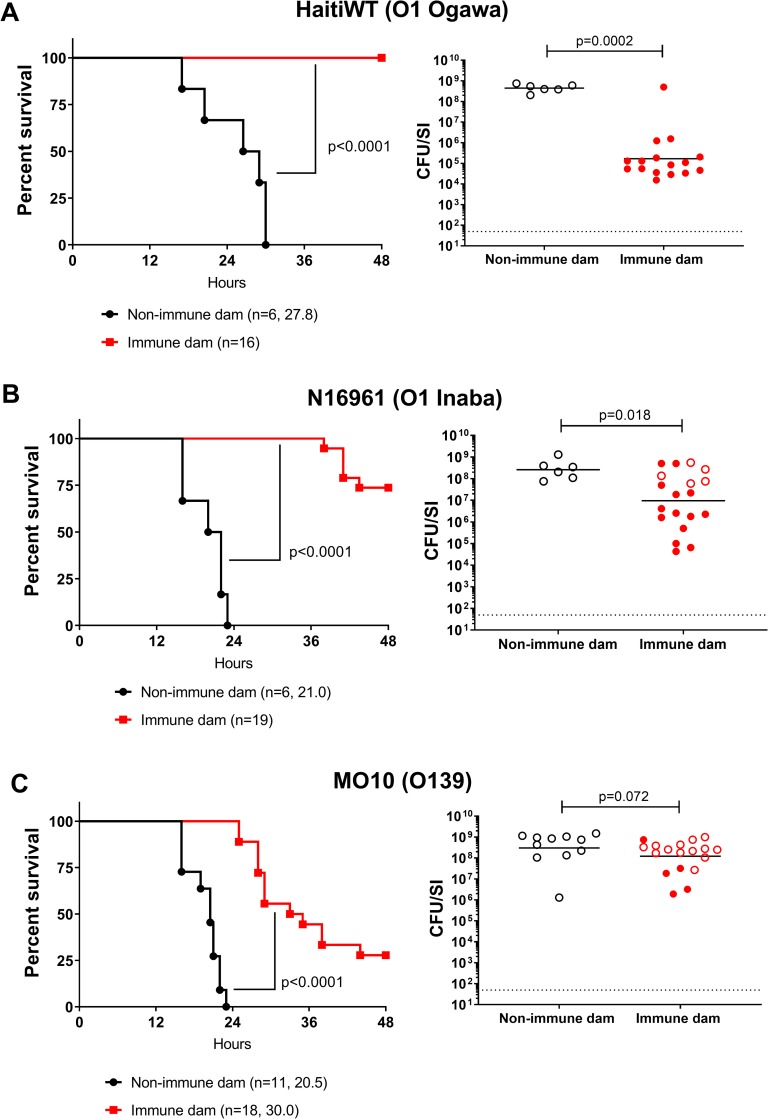
Survival of P3 C57BL/6 pups born to HaitiV-immunized or non-immunized dams after challenge with different serotype and serogroup *V*. *cholerae* strains. Pups born to either HaitiV-immunized dams (red) or non-immunized SPF dams (black) were challenged with either the O1 Ogawa strain HaitiWT (A), the O1 Inaba strain N16961 (B) or the O139 strain MO10 (C). Panels on the left show survival curves for infected pups up to 48 hours post inoculation with the indicated strains; the number of pups challenged and the median survival time in hours is shown. P-values were determined by the Mantel-Cox method. Panels on the right show the intestinal WT *V*. *cholerae* burden in pups at the time of death (open circles) or at 48 hpi (closed circles). Burden is plotted as CFU/SI. The dotted line marks the limit of detection (50 CFU/SI). P-values were determined by the Mann-Whitney U test.

We next mated HaitiV-immunized animals from cohort 2 with age-matched GF male mice, thereby preserving their colonization with HaitiV. When challenged with HaitiWT, none of the 16 pups born to HaitiV-immunized dams developed signs of diarrhea or died by 48 hpi; in stark contrast, all pups born to non-immunized dams died within ~30 hpi (Fig 6A, left). There was a marked ~5,000-fold reduction in the intestinal load of HaitiWT in pups born to immunized versus control dams (Fig 6A, right). The pups of the immunized dams remained healthy for at least 2 weeks post-challenge, even though there were still detectable but very low levels of HaitiWT in their intestinal homogenates (S1 Fig). Thus, oral immunization with HaitiV elicits an immune response that provides potent protection in nursing pups from diarrheal disease, death and *V*. *cholerae* intestinal colonization.

Pups of HaitiV-immunized dams were similarly challenged with heterologous *V*. *cholerae* strains, to test the serotype and serogroup specificity of protection engendered by oral immunization with HaitiV. The additional challenge strains included an O1 Inaba strain (N16961) that has been used as the challenge strain in several human volunteer cholera studies [43,46] and the serogroup O139 strain MO10, which was isolated during the 1992 O139 outbreak in India. Most pups from HaitiV-immunized dams were protected from N16961 *V*. *cholerae* challenge (7/10 survival at 48 hpi, Fig 6B). Despite the clinical protection, there was a much less dramatic reduction in the intestinal burden of N16961 (~20-fold) compared to that observed with HaitiWT challenge, indicating serotype-specific responses play an important role in limiting colonization. Surprisingly, pups challenged with MO10 also exhibited some protection, but there was no concomitant reduction in the intestinal burden of this O139 strain (Fig 6C). Together, these observations demonstrate that animals can exhibit protection from death despite relatively robust colonization, suggesting that protection from disease may result from immunity targeting factors such as CtxB, in addition to those that impede colonization.

### Pups fostered by HaitiV-immunized dams are protected from lethal *V*. *cholerae* challenge

Since our earlier studies indicated that HaitiV itself can mediate rapid protection against cholera independent of an adaptive immune response, it was important to investigate whether pups nursed by HaitiV immunized dams were colonized with the vaccine strain. Extensive plating of intestinal samples from the >50 pups used for survival assays (limit of detection = 50 CFU/small bowel) did not reveal any HaitiV CFU in the pups reared by HaitiV-shedding dams. Thus, vaccine strain transmission and its probiotic effects are almost certainly not the explanation for the potent protection observed in nursing pups.

**Fig 7:**
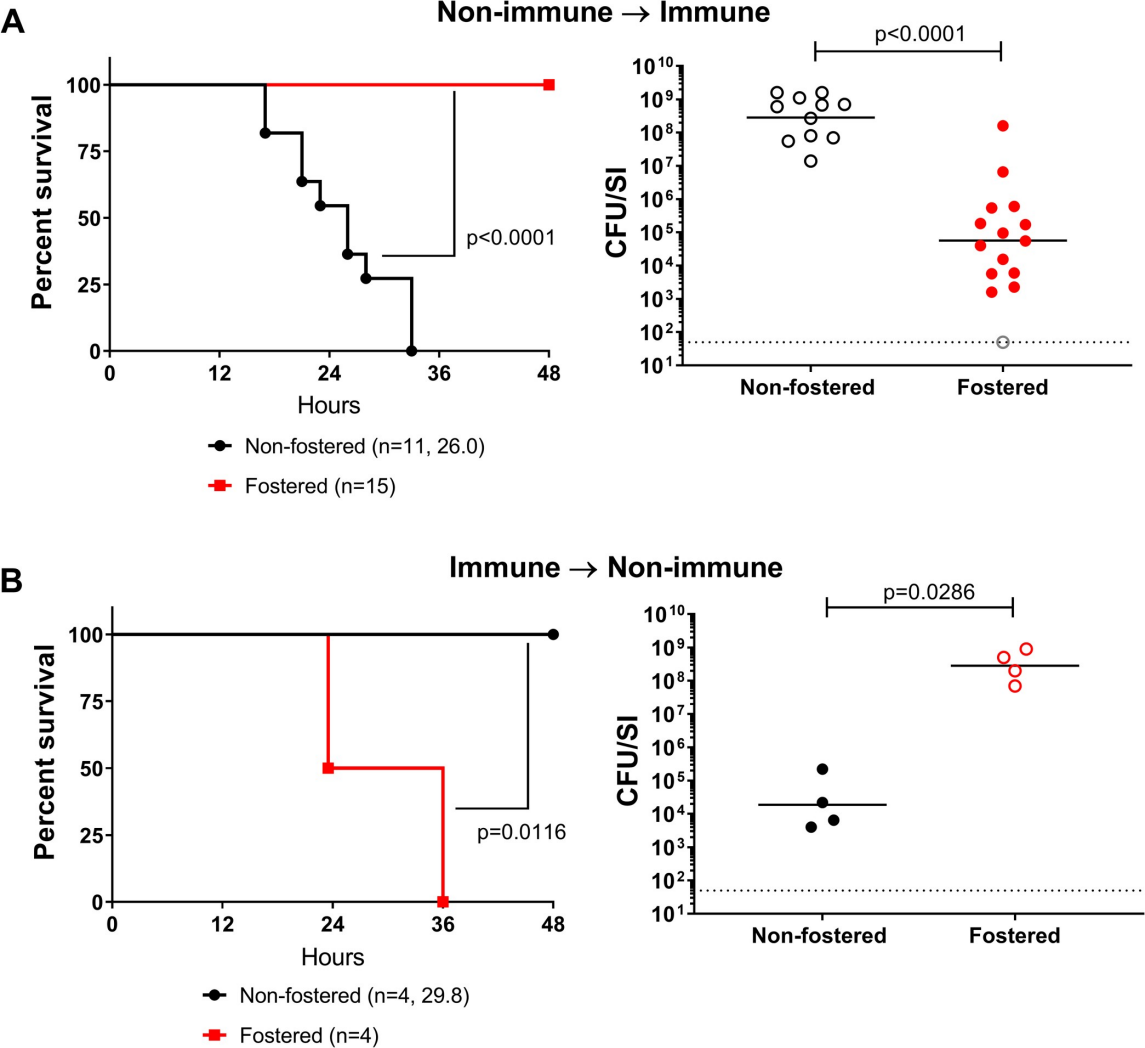
Survival of pups cross-fostered by HaitiV-immunized or non-immunized dams after challenge with HaitiWT. (A) Survival and *V*. *cholerae* intestinal colonization in pups born to non-immunized dams fostered by HaitiV-immunized dams. (B) Survival and *V*. *cholerae* intestinal colonization in pups born to HaitiV-immunized dams and fostered by non-immunized dams. Panel labeling, and statistics are as described in Fig 6. The gray open circle indicates a pup that had a CFU burden lower than the limit of detection.

Cross-fostering experiments were undertaken to investigate the likely passive nature of the protection. P1 pups born to SPF dams were transferred to and reared by HaitiV-immunized dams and then challenged 2 days later with HaitiWT (Fig 5A, between P1-P3). All pups crossed-fostered by immunized dams were protected (100% survival at 48 hpi) and nearly all had marked reductions (~1,000 fold) in their intestinal HaitiWT burdens (Fig 7A). These observations mirror the challenge studies presented above (Fig 6A), indicating passive immunity from milk accounts for the protection that HaitiV-immunized dams bestow to their progeny. Conversely, when pups born to HaitiV-immunized dams were cross-fostered by SPF (non-vaccinated) dams, all succumbed to HaitiWT challenge, albeit with an increase in median survival time (by ~6-hour) and had high HaitiWT intestinal burdens (Fig 7B). The modest extension in survival time in these mice may be due to trans-placentally derived immunity or residual milk from the HaitiV-immunized dam.

### A single oral dose of HaitiV is sufficient to elicit protective immunity

Although prior work with OCVs in GF mice suggested that multiple boosts were required to maximally induce immune responses, our observation of the prolonged colonization in multiply-vaccinated animals led us to test whether a single oral dose of HaitiV could also stimulate protective immune responses [37]. A singly-vaccinated group of female GF C57BL/6 mice (n = 4) was established to investigate this possibility. Like our studies with the multi-dose regimen, a single dose of HaitiV led to sustained colonization in the mice (S2 Fig). HaitiV induced vibriocidal antibody titers comparable in magnitude to those from serially immunized mice (Fig 2C). Litters from singly-immunized mice were also completely protected from disease resulting from HaitiWT challenge, phenocopying pups from the first cohorts (S2 Fig).

## Discussion

Vaccines for cholera are being increasingly embraced as public health tools for prevention of endemic cholera and limiting the spread of cholera epidemics [17]. Killed OCVs are efficacious in endemic populations, but live OCVs promise to be more potent, particularly in young children [19]. Here, we showed that the live OCV candidate HaitiV induces vibriocidal antibodies and other immunological correlates of protection against cholera in GF mice and leads to protection against disease in their offspring. Protection in this model was dependent on passively acquired factors in the milk of immunized dams and not transmission or colonization of HaitiV. Although our relatively small cohort sizes precluded rigorous statistical comparisons of immune responses in the immunized mice, oral administration of even a single dose of HaitiV elicited detectable vibriocidal antibodies in all animals. These observations provide strong data establishing HaitiV’s immunogenicity. Additionally, the comparable vibriocidal titers elicited by HaitiV and CVD-103HgR, a live OCV licensed by the FDA for travelers, bodes well for HaitiV’s immunogenicity in humans. Combining the immunogenicity data presented here with our finding that HaitiV can protect from cholera prior to the induction of adaptive immunity [29], suggests that HaitiV may function as a dual-acting agent, providing both rapid-onset short-term protection from disease while eliciting stable and long-lasting immunity against cholera.

Data from the challenge experiments (Fig 6) are consistent with the prevailing notion that serogroup, and to a lesser extent serotype are major determinants of protection against *V*. *cholerae* challenge [39,47]. Although it is thought that exposure to Inaba strains is more cross-protective than exposure to Ogawa strains, the relative potency of Inaba versus Ogawa vaccines in eliciting dual protection against both O1 serotypes requires further definition, as it has been suggested that both Ogawa and Inaba vaccine strains are good candidates for development [4–7]. A mixture of Ogawa and Inaba serotypes either as distinct strains or one bivalent strain (serotype Hikojima) may be beneficial in broadening the breadth of the immune response to HaitiV [48,49].

The modest protection that HaitiV immunization provided against *V*. *cholerae* O139 was unexpected. The epidemiology of the original O139 outbreak and experimental studies in rabbits demonstrate a lack of cross-protection between the two serogroups [14,15,39]. Notably, although pups born to HaitiV-immunized dams and challenged with MO10 survived longer than pups born to non-immunized dams, there was little difference in the MO10 intestinal colonization between these groups (Fig 6C). The discrepancy between clinical protection and relatively robust colonization suggests that HaitiV stimulates immune responses to *V*. *cholerae* factors, like CT, that may contribute to disease but not directly to colonization. The capacity of live OCVs to induce immune responses to in vivo-expressed antigens, including CtxB, is a property that heightens the appeal of live vs killed OCVs [27,50].

Although GF mice enabled us to test the protective efficacy of a candidate live OCV, their absence of the microbiota and resulting improper immune development are important caveats to consider. The GF model does not recapitulate the competitive microbial environment that live OCVs will encounter in the human host. We observed similar prolonged shedding patterns for both CVD-103HgR and HaitiV in the GF mice (Fig 1), yet CVD-103HgR is known to be shed by human volunteers at a low frequency for a short period [25,51]. Thus, our findings likely overestimate HaitiV’s capacity to colonize the human intestine. The observation that exposure of HaitiV-immunized dams to SPF-derived pups during the cross-fostering experiments led to the elimination of detectable HaitiV in feces supports the prediction that this vaccine will not stably colonize humans. Since we employed a multi-dose vaccination schedule here, it remains an open question whether transient exposure of naïve mice to HaitiV will also stimulate protective immunity, as has been shown in the context of vaccination with *V*. *cholerae* outer membrane vesicles [52,53]. The streptomycin-treated mouse model of *V*. *cholerae* colonization, which allows for temporary intestinal colonization, may also be useful to investigate the duration of colonization required for immunity [54]. Ultimately, the capacity of HaitiV to colonize the intestine and the relationship between colonization and protective immunity will need to be defined in human volunteers.

The immunogenicity of live OCVs in mice has only been investigated in GF animals because adult mice with intact microbiota are refractory to intestinal *V*. *cholerae* replication and colonization. However, previous studies of live OCVs in GF mice only analyzed immune correlates of protection and not protection against challenge [36,37]. The combination of the neonatal survival assay with the oral GF vaccination model builds on existing knowledge of these mice to assay both the immunogenicity and protective efficacy of live OCV candidates [38]. This model may be a useful addition to existing approaches that probe the molecular bases of vaccine-mediated mucosal protection against pathogens, a topic with significant translational potential that remains poorly understood [53,55]. A recent report employing a similar maternal-infant transmission model in the context of intraperitoneally-delivered heat-killed *Citrobacter rodentium* highlights the versatility of assessing vaccine protective efficacy using the infant progeny of immunized animals as readouts [56]. The broad availability of genetically engineered mice and the relative ease of GF-derivation provides a powerful opportunity to leverage both host and bacterial genetics to explore how live-OCVs can be optimized to better defend against this ancient pathogen.

## Supporting information

S1 Fig Duration of protection from HaitiWT in pups from HaitiV-immunized dams. Pups were inoculated identically to initial challenge studies and allowed to age normally for up to 14 days (336 hours) before enumeration of intestinal burdens. The gray open circle represents an intestinal burden that was below the limit of detection (dotted line).
(TIF)

S2 Fig Effectiveness of a single-dose HaitiV vaccination regimen in C57BL/6 mice. (A): Fecal shedding of HaitiV from mice given a single dose of HaitiV at Day 0. (B): Vibriocidal antibody titers from singly-immunized mice against either Ogawa (white) or Inaba (gray) target strains. (C): Survival (left) and intestinal colonization (right) of pups from singly-immunized dams challenged with a lethal dose of HaitiWT. The dotted line marks the limit of detection.
(TIF)

S1 Table Bacterial strains used in this study. ET = El Tor, VET = Variant El Tor, Km = kanamycin, Sm = streptomycin, SXT = sulfamethoxazole/trimethoprim, R = resistant, S = sensitive.
(DOCX)

S2 Table Multiple comparisons testing of antibody titers in cohort 2. P-values shown were calculated from a Dunnett’s multiple-comparison test comparing Day 14, 28 or 42 mean titers to the mean titer at Day 7. P-values are shown to three significant figures and values < 0.05 are bolded.
(DOCX)

S1 References Supplemental references for S1 Table. (DOCX)

## References

[1] Ali M, Nelson AR, Lopez AL, Sack DA. Updated global burden of cholera in endemic countries. PLoS Negl Trop Dis. 2015;9: 1–13. 10.1371/journal.pntd.0003832 26043000 PMC4455997

[2] Clemens JD, Nair GB, Ahmed T, Qadri F, Holmgren J. Cholera. Lancet (London, England). 2017;6736: 1–11. 10.1016/S0140-6736(17)30559-7 28302312

[3] Hisatsune K, Kondo S, Isshiki Y, Iguchi T, Haishima Y. Occurrence of 2-O-Methyl-N-(3-Deoxy-L-glycero-tetronyl)-D-perosamine (4-amino-4,6-dideoxy-D-manno-pyranose) in Lipopolysaccharide from Ogawa but Not from Inaba O Forms of O1 Vibrio cholerae. Biochemical and Biophysical Research Communications. 1993 pp. 302–307. 10.1006/bbrc.1993.1046 8422256

[4] Longini IM Jr., Yunus M, Zaman K, Siddique AK, Sack RB, Nizam A. Epidemic and Endemic Cholera Trends over a 33‐Year Period in Bangladesh. J Infect Dis. 2002;186: 246–251. 10.1086/341206 12134262

[5] Mosley WH, Aziz KMA, Rahman AS, Chowdhury AK, Ahmed A. Field trials of monovalent Ogawa and Inaba cholera vaccines in rural Bangladesh—three years of observation. Bull World Health Organ. 1973;49: 381–7. 4604478 PMC2480950

[6] Khan AI, Chowdhury F, Harris JB, Larocque RC, Faruque ASG, Ryan ET, et al Comparison of clinical features and immunological parameters of patients with dehydrating diarrhoea infected with Inaba or Ogawa serotypes of Vibrio cholerae O1. Scand J Infect Dis. 2010;42: 48–56. 10.3109/00365540903289688 19883159 PMC3786431

[7] Ali M, Emch M, Park JK, Yunus M, Clemens J. Natural cholera infection-derived immunity in an endemic setting. J Infect Dis. 2011;204: 912–918. 10.1093/infdis/jir416 21849288 PMC3156915

[8] Mutreja A, Kim DW, Thomson NR, Connor TR, Lee JH, Kariuki S, et al Evidence for several waves of global transmission in the seventh cholera pandemic. Nature. 2011;477: 462–5. 10.1038/nature10392 21866102 PMC3736323

[9] Nair GB, Faruque SM, Bhuiyan NA, Kamruzzaman M, Siddique AK, Sack DA. New variants of Vibrio cholerae O1 biotype El Tor with attributes of the classical biotype from hospitalized patients with acute diarrhea in Bangladesh. J Clin Microbiol. 2002;40: 3296–9. 10.1128/JCM.40.9.3296-3299.2002 12202569 PMC130785

[10] Chin C-S, Sorenson J, Harris JB, Robins WP, Charles RC, Jean-Charles RR, et al The origin of the Haitian cholera outbreak strain. N Engl J Med. 2011;364: 33–42. 10.1056/NEJMoa1012928 21142692 PMC3030187

[11] Nair GB, Qadri F, Holmgren J, Svennerholm AM, Safa A, Bhuiyan NA, et al Cholera due to altered El Tor strains of Vibrio cholerae O1 in Bangladesh. J Clin Microbiol. 2006;44: 4211–4213. 10.1128/JCM.01304-06 16957040 PMC1698305

[12] Safa A, Sultana J, Cam PD, Mwansa JC, Kong RYC. Vibrio cholerae O1 hybrid El Tor Strains, Asia and Africa. Emerg Infect Dis. 2008;14: 987–988. 10.3201/eid1406.080129 18507925 PMC2600311

[13] Chowdhury F, Mather AE, Begum YA, Asaduzzaman M, Baby N, Sharmin S, et al Vibrio cholerae Serogroup O139: Isolation from Cholera Patients and Asymptomatic Household Family Members in Bangladesh between 2013 and 2014. PLoS Negl Trop Dis. 2015;9: 1–11. 10.1371/journal.pntd.0004183 26562418 PMC4642977

[14] Albert MJ, Alam K, Ansaruzzaman M, Qadri F, Sack RB. Lack of cross-protection against diarrhea due to Vibrio cholerae O139 (Bengal strain) after oral immunization of rabbits with V. cholerae O1 vaccine strain CVD103-HgR. J Infect Dis. 1994;169: 230–1. 10.1093/infdis/169.1.230 8277193

[15] Albert MJ, Alam K, Rahman AS, Huda S, Sack RB. Lack of cross-protection against diarrhea due to Vibrio cholerae O1 after oral immunization of rabbits with V. cholerae O139 Bengal. J Infect Dis. 1994;169: 709–10. 10.1093/infdis/169.3.709 8158063

[16] Qadri F, Wennerås C, Albert MJ, Hossain J, Mannoor K, Begum YA, et al Comparison of immune responses in patients infected with Vibrio cholerae O139 and O1. Infect Immun. 1997;65: 3571–3576. 9284121 10.1128/iai.65.9.3571-3576.1997 PMC175508

[17] World Health Organization. Cholera vaccines: WHO position paper—August 2017. Wkly Epidemiol Rec. 2017;No 34: 477–500. 10.1016/S0140-6736(09)61877

[18] Ali M, Emch M, Von Seidlein L, Yunus M, Sack DA, Rao M, et al Herd immunity conferred by killed oral cholera vaccines in Bangladesh: A reanalysis. Lancet. 2005;366: 44–49. 10.1016/S0140-6736(05)66550-6 15993232

[19] Bi Q, Ferreras E, Pezzoli L, Legros D, Ivers LC, Date K, et al Protection against cholera from killed whole-cell oral cholera vaccines: a systematic review and meta-analysis. Lancet Infect Dis. 2017;17: 1080–1088. 10.1016/S1473-3099(17)30359-6 28729167 PMC5639147

[20] Qadri F, Ali M, Lynch J, Chowdhury F, Khan AI, Wierzba TF, et al Efficacy of a single-dose regimen of inactivated whole-cell oral cholera vaccine: results from 2 years of follow-up of a randomised trial. Lancet Infect Dis. 2018;18: 666–674. 10.1016/S1473-3099(18)30108-7 29550406

[21] Leung DT, Rahman MA, Mohasin M, Patel SM, Aktar A, Khanam F, et al Memory B cell and other immune responses in children receiving two doses of an oral killed cholera vaccine compared to responses following natural cholera infection in Bangladesh. Clin Vaccine Immunol. 2012;19: 690–698. 10.1128/CVI.05615-11 22441386 PMC3346319

[22] Azman AS, Parker LA, Rumunu J, Tadesse F, Grandesso F, Deng LL, et al Effectiveness of one dose of oral cholera vaccine in response to an outbreak: a case-cohort study. Lancet Glob Heal. 2016;4: e856–e863. 10.1016/S2214-109X(16)30211-X 27765293

[23] Qadri F, Wierzba TF, Ali M, Chowdhury F, Khan AI, Saha A, et al Efficacy of a Single-Dose, Inactivated Oral Cholera Vaccine in Bangladesh. N Engl J Med. 2016;374: 1723–32. 10.1056/NEJMoa1510330 27144848

[24] Levine MM, Chen WH, Kaper JB, Lock M, Danzig L, Gurwith M. PaxVax CVD 103-HgR single-dose live oral cholera vaccine. Expert Rev Vaccines. 2017;16: 197–213. 10.1080/14760584.2017.1291348 28165831

[25] Lagos R, San Martin O, Wasserman SS, Prado V, Losonsky GA, Bustamante C, et al Palatability, reactogenicity and immunogenicity of engineered live oral cholera vaccine CVD 103-HgR in Chilean infants and toddlers. Pediatr Infect Dis J. 1999;18: 624–30. 10440439 10.1097/00006454-199907000-00011

[26] Qadri F, Chowdhury MI, Faruque SM, Salam MA, Ahmed T, Begum YA, et al Peru-15, a live attenuated oral cholera vaccine, is safe and immunogenic in Bangladeshi toddlers and infants. Vaccine. 2007;25: 231–8. 10.1016/j.vaccine.2006.08.031 16996172

[27] Hang L, John M, Asaduzzaman M, Bridges EA, Vanderspurt C, Kirn TJ, et al Use of in vivo-induced antigen technology (IVIAT) to identify genes uniquely expressed during human infection with Vibrio cholerae. Proc Natl Acad Sci U S A. 2003;100: 8508–13. 10.1073/pnas.1431769100 12826608 PMC166259

[28] Tacket CO, Losonsky G, Nataro JP, Cryz SJ, Edelman R, Kaper JB, et al Onset and duration of protective immunity in challenged volunteers after vaccination with live oral cholera vaccine CVD 103-HgR. J Infect Dis. 1992;166: 837–41. 10.1093/infdis/166.4.837 1527420

[29] Hubbard TP, Billings G, Dörr T, Sit B, Warr AR, Kuehl CJ, et al A live vaccine rapidly protects against cholera in an infant rabbit model. Sci Transl Med. 2018;10 10.1126/scitranslmed.aap8423 29899024 PMC6500431

[30] Ritchie JM, Rui H, Bronson RT, Waldor MK. Back to the future: studying cholera pathogenesis using infant rabbits. MBio. 2010;1: 1–13. 10.1128/mBio.00047-10 20689747 PMC2912669

[31] Rollenhagen JE, Kalsy A, Cerda F, John M, Harris JB, LaRocque RC, et al Transcutaneous immunization with toxin-coregulated pilin A induces protective immunity against Vibrio cholerae O1 El Tor challenge in mice. Infect Immun. 2006;74: 5834–5839. 10.1128/IAI.00438-06 16988262 PMC1594919

[32] Alam MM, Bufano MK, Xu P, Kalsy A, Yu Y, Freeman YW, et al Evaluation in mice of a conjugate vaccine for cholera made from Vibrio cholerae O1 (Ogawa) O-specific polysaccharide. PLoS Negl Trop Dis. 2014;8: e2683 10.1371/journal.pntd.0002683 24516685 PMC3916310

[33] Tarique AA, Kalsy A, Arifuzzaman M, Rollins SM, Charles RC, Leung DT, et al Transcutaneous immunization with a Vibrio cholerae O1 Ogawa synthetic hexasaccharide conjugate following oral whole-cell cholera vaccination boosts vibriocidal responses and induces protective immunity in mice. Clin Vaccine Immunol. 2012;19: 594–602. 10.1128/CVI.05689-11 22357651 PMC3318280

[34] Sayeed MA, Bufano MK, Xu P, Eckhoff G, Charles RC, Alam MM, et al A Cholera Conjugate Vaccine Containing O-specific Polysaccharide (OSP) of V. cholerae O1 Inaba and Recombinant Fragment of Tetanus Toxin Heavy Chain (OSP:rTTHc) Induces Serum, Memory and Lamina Proprial Responses against OSP and Is Protective in Mice. PLoS Negl Trop Dis. 2015;9: e0003881 10.1371/journal.pntd.0003881 26154421 PMC4495926

[35] Sasaki S, Onishi N, Suzuki R, Adachi K, Miyashita M, Shimamura T, et al The relation between the persistence of El Tor vibrio in the intestines of germfree mice and so-called coproantibody. J Infect Dis. 1970;121: Suppl 121:124+. 4316145 10.1093/infdis/121.supplement.s124

[36] Butterton JR, Ryan ET, Shahin RA, Calderwood SB. Development of a germfree mouse model of Vibrio cholerae infection. Infect Immun. 1996;64: 4373–7. 8926115 10.1128/iai.64.10.4373-4377.1996 PMC174383

[37] Crean TI, John M, Calderwood SB, Ryan ET. Optimizing the germfree mouse model for in vivo evaluation of oral Vibrio cholerae vaccine and vector strains. Infect Immun. 2000;68: 977–81. 10.1128/iai.68.2.977-981.2000 10639476 PMC97235

[38] Shimamura T, Tazume S, Hashimoto K, Sasaki S. Experimental cholera in germfree suckling mice. Infect Immun. 1981;34: 296–8. 7298190 10.1128/iai.34.1.296-298.1981 PMC350856

[39] Harris JB. Cholera: Immunity and Prospects in Vaccine Development. J Infect Dis. 2018;218: S141–S146. 10.1093/infdis/jiy414 30184117 PMC6188552

[40] Sow SO, Tapia MD, Chen WH, Haidara FC, Kotloff KL, Pasetti MF, et al A randomized, placebo-controlled, double-blind Phase 2 trial comparing the reactogenicity and immunogenicity of a single ≥2x108 colony forming units [cfu] standard-dose versus a ≥2x109 cfu high-dose of CVD 103-HgR live attenuated oral cholera vaccine, with Shanchol as an open-label immunologic comparator. Clin Vaccine Immunol. 2017;24: e00265–17. 10.1128/CVI.00265-17 29021299 PMC5717191

[41] Qadri F, Chowdhury MI, Faruque SM, Salam MA, Ahmed T, Begum YA, et al Randomized, controlled study of the safety and immunogenicity of Peru-15, a live attenuated oral vaccine candidate for cholera, in adult volunteers in Bangladesh. J Infect Dis. 2005;192: 573–9. 10.1086/432074 16028125

[42] Haney DJ, Lock MD, Simon JK, Harris J, Gurwith M. Antibody-Based Correlates of Protection Against Cholera Analysis of a Challenge Study in a Cholera-Naïve Population. Clin Vaccine Immunol. 2017;24: e00098–17. 10.1128/CVI.00098-17 28566334 PMC5583470

[43] Chen WH, Cohen MB, Kirkpatrick BD, Brady RC, Galloway D, Gurwith M, et al Single-dose Live Oral Cholera Vaccine CVD 103-HgR Protects Against Human Experimental Infection With Vibrio cholerae O1 El Tor. Clin Infect Dis. 2016;62: 1329–1335. 10.1093/cid/ciw145 27001804 PMC4872293

[44] Chaicumpa W, Rowley D. Experimental cholera in infant mice: protective effects of antibody. J Infect Dis. 1972;125: 480–5. 10.1093/infdis/125.5.480 5023642

[45] Ujiye A, Nakatomi M, Utsunomiya A, Mitsui K, Sogame S, Iwanaga M, et al Experimental Cholera in Mice. I. First Report on the Oral Infection. Trop Med. 1968;10: 65–71.

[46] Cohen MB, Giannella RA, Bean J, Taylor DN, Parker S, Hoeper A, et al Randomized, controlled human challenge study of the safety, immunogenicity, and protective efficacy of a single dose of Peru-15, a live attenuated oral cholera vaccine. Infect Immun. 2002;70: 1965–70. 10.1128/IAI.70.4.1965-1970.2002 11895960 PMC127885

[47] Islam K, Hossain M, Kelly M, Mayo Smith LM, Charles RC, Bhuiyan TR, et al Anti-O-specific polysaccharide (OSP) immune responses following vaccination with oral cholera vaccine CVD 103-HgR correlate with protection against cholera after infection with wild-type Vibrio cholerae O1 El Tor Inaba in North American volunteers. PLoS Negl Trop Dis. 2018;12: e0006376 10.1371/journal.pntd.0006376 29624592 PMC5906022

[48] Alam MT, Ray SS, Chun CN, Chowdhury ZG, Rashid MH, Madsen Beau De Rochars VE, et al Major Shift of Toxigenic V. cholerae O1 from Ogawa to Inaba Serotype Isolated from Clinical and Environmental Samples in Haiti. PLoS Negl Trop Dis. 2016;10: 1–9. 10.1371/journal.pntd.0005045 27716803 PMC5055329

[49] Sack RB, Miller CE. Progressive changes of Vibrio serotypes in germ-free mice infected with Vibrio cholerae. J Bacteriol. 1969;99: 688–695. 5370274 10.1128/jb.99.3.688-695.1969 PMC250082

[50] Charles RC, Nakajima R, Liang L, Jasinskas A, Berger A, Leung DT, et al The plasma and mucosal IgM, IgA, and IgG responses to the Vibrio cholerae O1 protein immunome in adults with cholera in Bangladesh. J Infect Dis. 2017; 37–41. 10.1093/infdis/jix253 28535267 PMC5853614

[51] Tacket CO, Losonsky G, Nataro JP, Cryz SJ, Edelman R, Kaper JB, et al Onset and duration of protective immunity in challenged volunteers after vaccination with live oral cholera vaccine CVD 103-HgR. J Infect Dis. 1992;166: 837–41. 10.1093/infdis/166.4.837 1527420

[52] Bishop AL, Tarique AA, Patimalla B, Calderwood SB, Qadri F, Camilli A. Immunization of mice with vibrio cholerae outer-membrane vesicles protects against hyperinfectious challenge and blocks transmission. J Infect Dis. 2012;205: 412–21. 10.1093/infdis/jir756 22147790 PMC3256948

[53] Wang Z, Lazinski DW, Camilli A. Immunity provided by an outer membrane vesicle cholera vaccine is due to O-antigenspecific antibodies inhibiting bacterial motility. Infect Immun. 2017;85: 1–9. 10.1128/IAI.00626-16 27795359 PMC5203661

[54] Nygren E, Li B-L, Holmgren J, Attridge SR. Establishment of an adult mouse model for direct evaluation of the efficacy of vaccines against Vibrio cholerae. Infect Immun. 2009;77: 3475–84. 10.1128/IAI.01197-08 19470748 PMC2715679

[55] van Splunter M, van Hoffen E, Floris-Vollenbroek EG, Timmerman H, de Bos EL, Meijer B, et al Oral cholera vaccination promotes homing of IgA+ memory B cells to the large intestine and the respiratory tract. Mucosal Immunol. 2018;11: 1254–1264. 10.1038/s41385-018-0006-7 29467446

[56] Caballero-Flores G, Sakamoto K, Zeng MY, Wang Y, Hakim J, Matus-Acuña V, et al Maternal Immunization Confers Protection to the Offspring against an Attaching and Effacing Pathogen through Delivery of IgG in Breast Milk. Cell Host Microbe. 2019; 10.1016/j.chom.2018.12.015 30686564 PMC6375740

