# Supplementary figures and images for: Oral immunization with a probiotic cholera vaccine induces broad protective immunity against Vibrio cholerae colonization and disease in mice
Source: PLoS Negl Trop Dis. 2019 May 31;13(5):e0007417. doi: 10.1371/journal.pntd.0007417 (PMC6561597; doi:10.1371/journal.pntd.0007417)

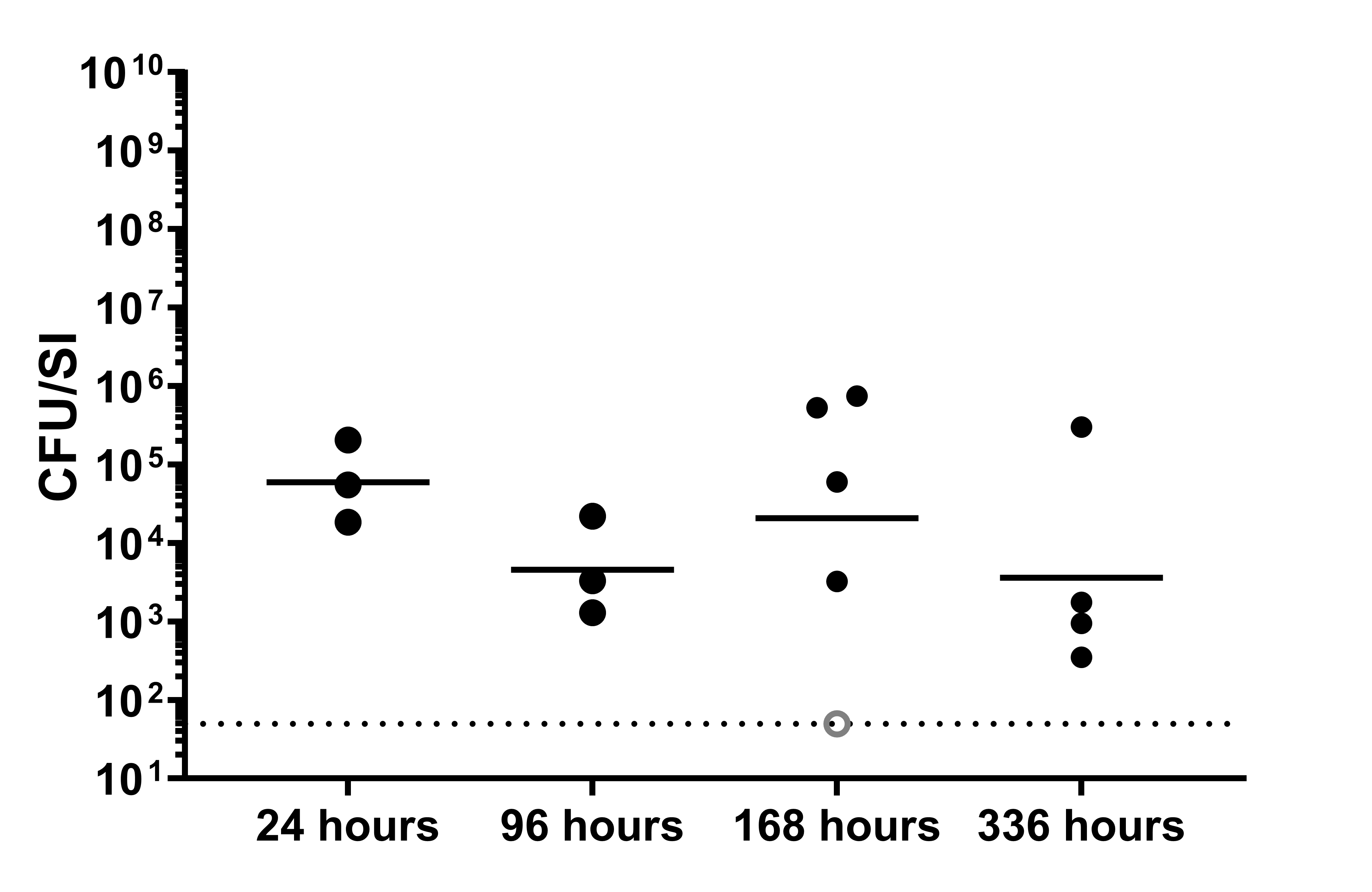

Supplement: S1 Fig — Pups were inoculated identically to initial challenge studies and allowed to age normally for up to 14 days (336 hours) before enumeration of intestinal burdens. The gray open circle represents an intestinal burden that was below the limit of detection (dotted line). (TIF) [file pntd.0007417.s001.tif]

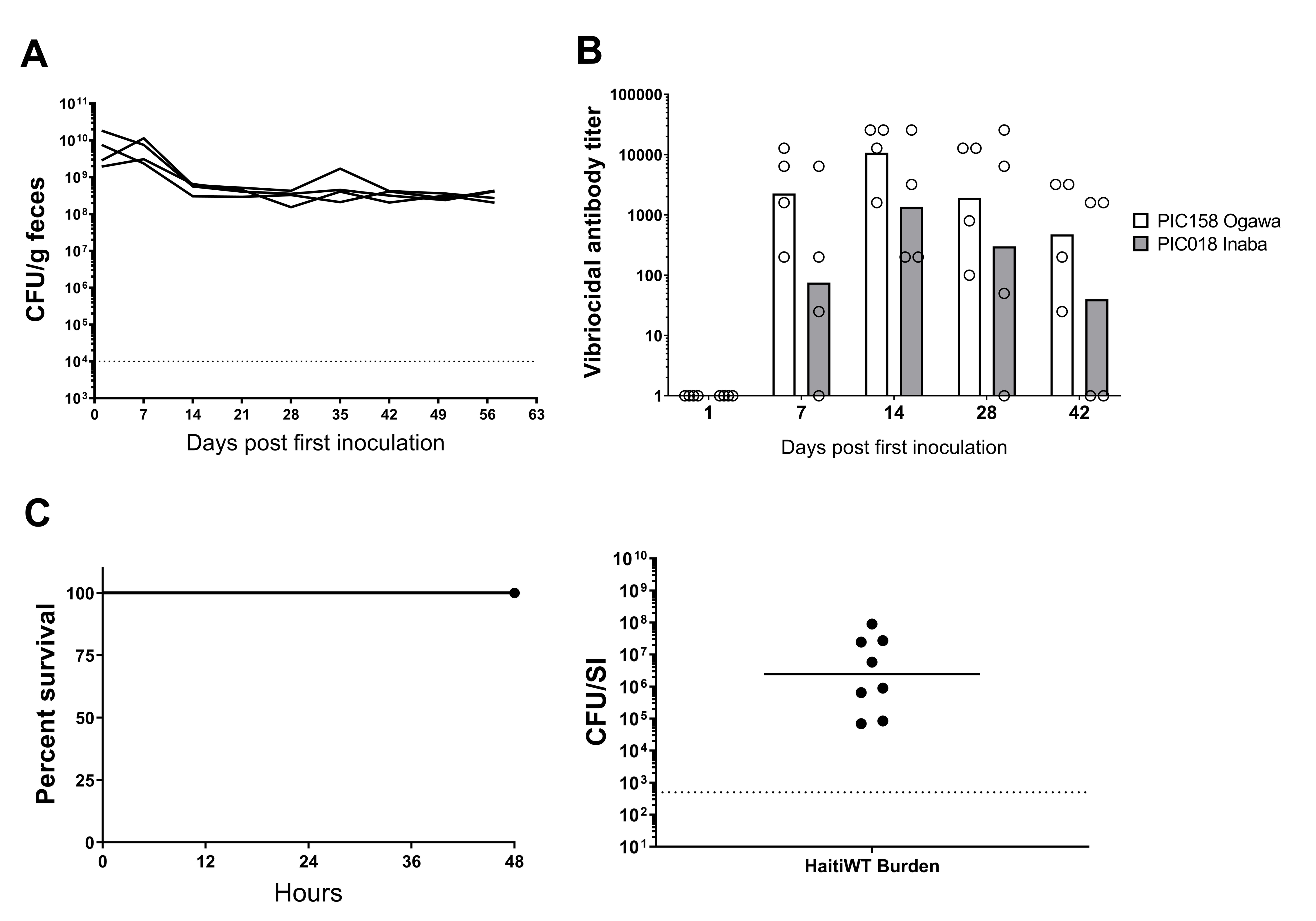

Supplement: S2 Fig — (A): Fecal shedding of HaitiV from mice given a single dose of HaitiV at Day 0. (B): Vibriocidal antibody titers from singly-immunized mice against either Ogawa (white) or Inaba (gray) target strains. (C): Survival (left) and intestinal colonization (right) of pups from singly-immunized dams challenged with a lethal dose of HaitiWT. The dotted line marks the limit of detection. (TIF) [file pntd.0007417.s002.tif]
