# Supplementary material for: Oral immunization with a probiotic cholera vaccine induces broad protective immunity against Vibrio cholerae colonization and disease in mice
Source: PLoS Negl Trop Dis. 2019 May 31;13(5):e0007417. doi: 10.1371/journal.pntd.0007417 (PMC6561597; doi:10.1371/journal.pntd.0007417)
Supplement: S1 Table — ET = El Tor, VET = Variant El Tor, Km = kanamycin, Sm = streptomycin, SXT = sulfamethoxazole/trimethoprim, R = resistant, S = sensitive. (DOCX) [file pntd.0007417.s003.docx]

| **Strain** | **Notes** | **Source** |
| --- | --- | --- |
| *E. coli* SM10λpir | Donor strain for *ctxAB* deletion, KmR | [29] |
| *V. cholerae* HaitiWT | O1 Ogawa VET, SmR SXTR, *lac+* | [10] |
| *V. cholerae* Haiti *ΔctxAB* | O1 Ogawa VET, SmR SXTR, *lac+* | This study |
| *V. cholerae* HaitiV | O1 Ogawa VET, SmR SXTS, *lac-, recA+* | [29] |
| *V. cholerae* CVD103-HgR | O1 Inaba Classical, SmR, *lac+* | This study |
| *V. cholerae* N16961 | O1 Inaba ET, SmR, *lac+* | [Supp1] |
| *V. cholerae* MO10 | O139, SXTR, *lac+* | [Supp2] |
| *V. cholerae* PIC018 | O1 Inaba ET | [34] |
| *V. cholerae* PIC158 | O1 Ogawa ET | [34] |
