# Supplementary material for: Oral immunization with a probiotic cholera vaccine induces broad protective immunity against Vibrio cholerae colonization and disease in mice
Source: PLoS Negl Trop Dis. 2019 May 31;13(5):e0007417. doi: 10.1371/journal.pntd.0007417 (PMC6561597; doi:10.1371/journal.pntd.0007417)
Supplement: S2 Table — P-values shown were calculated from a Dunnett’s multiple-comparison test comparing Day 14, 28 or 42 mean titers to the mean titer at Day 7. P-values are shown to three significant figures and values < 0.05 are bolded. (DOCX) [file pntd.0007417.s004.docx]

| **Sample** | **Day 14 vs. Day 7** | **Day 28 vs. Day 7** | **Day 42 vs. Day 7** |
| --- | --- | --- | --- |
| Inaba OSP IgA | 0.560 | 0.112 | **0.0197** |
| Inaba OSP IgG | 0.999 | 0.117 | 0.0579 |
| Inaba OSP IgM | **0.0157** | **0.0428** | **0.0357** |
| Ogawa OSP IgA | 0.531 | 0.0828 | 0.146 |
| Ogawa OSP IgG | 0.550 | **0.0205** | **0.0172** |
| Ogawa OSP IgM | 0.0630 | **0.0253** | **0.0343** |
| CtxB IgA | 0.750 | 0.571 | 0.207 |
| CtxB IgG | 0.694 | 0.263 | 0.112 |
| CtxB IgM | 0.267 | **0.0498** | **0.013** |
