# Supplementary material for: Oral immunization with a probiotic cholera vaccine induces broad protective immunity against Vibrio cholerae colonization and disease in mice
Source: PLoS Negl Trop Dis. 2019 May 31;13(5):e0007417. doi: 10.1371/journal.pntd.0007417 (PMC6561597; doi:10.1371/journal.pntd.0007417)
Supplement: S1 References — (DOCX) [file pntd.0007417.s005.docx]

1. Heidelberg JF, Eisen JA, Nelson WC, Clayton RA, Gwinn ML, Dodson RJ, et al. DNA sequence of both chromosomes of the cholera pathogen Vibrio cholerae. Nature. 2000;406: 477–83. doi:10.1038/35020000

2. Waldor MK, Mekalanos JJ. Emergence of a new cholera pandemic: molecular analysis of virulence determinants in Vibrio cholerae O139 and development of a live vaccine prototype. J Infect Dis. 1994;170: 278–83.
